# Beta-Boswellic Acid Reverses 3-Nitropropionic Acid-Induced Molecular, Mitochondrial, and Histopathological Defects in Experimental Rat Model of Huntington’s Disease

**DOI:** 10.3390/biomedicines10112866

**Published:** 2022-11-09

**Authors:** Thamer H. Albekairi, Arzoo Kamra, Sudeep Bhardwaj, Sidharth Mehan, Aditi Giri, Manisha Suri, Abdulrahman Alshammari, Metab Alharbi, Abdullah F. Alasmari, Acharan S Narula, Reni Kalfin

**Affiliations:** 1 Department of Pharmacology and Toxicology, College of Pharmacy, King Saud University, P.O. Box 2455, Riyadh 11451, Saudi Arabia; 2 Department of Pharmacology, Seth G.L. Bihani S.D. College of Technical Education, Institute of Pharmaceutical Sciences and Drug Research, Sri Ganganagar 335001, Rajasthan, India; 3 Division of Neuroscience, Department of Pharmacology, ISF College of Pharmacy (An Autonomous College), Moga 142001, Punjab, India; 4 Narula Research, LLC, 107 Boulder Bluff, Chapel Hill, NC 27516, USA; 5 Institute of Neurobiology, Bulgarian Academy of Sciences, Acad. G. Bonchev St., Block 23, 1113 Sofia, Bulgaria; 6 Department of Healthcare, South-West University “NeofitRilski”, Ivan Mihailov St. 66, 2700 Blagoevgrad, Bulgaria

**Keywords:** β-Boswellic acid (β-BA), Huntington’s disease (HD), mitochondrial ETC-complexes, 3-nitropropionic acid (3-NP), neuroprotection

## Abstract

Huntington’s disease (HD) is distinguished by a triple repeat of CAG in exon 1, an increase in poly Q in the Htt gene, and a loss of GABAergic medium spiny neurons (MSN) in the striatum and white matter of the cortex. Mitochondrial ETC-complex dysfunctions are involved in the pathogenesis of HD, including neuronal energy loss, synaptic neurotrophic decline, neuronal inflammation, apoptosis, and grey and white matter destruction. A previous study has demonstrated that beta Boswellic acid (β-BA), a naturally occurring phytochemical, has several neuroprotective properties that can reduce pathogenic factors associated with various neurological disorders. The current investigation aimed to investigate the neuroprotective potential of β-BA at oral doses of 5, 10, and 15 mg/kg alone, as well as in conjunction with the potent antioxidant vitamin E (8 mg/kg, orally) in 3-NP-induced experimental HD rats. Adult Wistar rats were separated into seven groups, and 3-NP, at a dose of 10 mg/kg, was orally administered to each group of adult Wistar rats beginning on day 1 and continuing through day 14. The neurotoxin 3-NP induces neurodegenerative, g, neurochemical, and pathological alterations in experimental animals. Continuous injection of 3-NP, according to our results, aggravated HD symptoms by suppressing ETC-complex-II, succinate dehydrogenase activity, and neurochemical alterations. β-BA, when taken with vitamin E, improved behavioural dysfunctions such as neuromuscular and motor impairments, as well as memory and cognitive abnormalities. Pharmacological treatments with β-BA improved and restored ETC complexes enzymes I, II, and V levels in brain homogenates. β-BA treatment also restored neurotransmitter levels in the brain while lowering inflammatory cytokines and oxidative stress biomarkers. β-BA’s neuroprotective potential in reducing neuronal death was supported by histopathological findings in the striatum and cortex. As a result, the findings of this research contributed to a better understanding of the potential role of natural phytochemicals β-BA in preventing neurological illnesses such as HD.

## 1. Introduction

Huntington’s disease (HD) is a fatal neurological disease that is autosomal, dominantly inherited, fully penetrant, and characterized by progressive chorea, motor and mental issues, cognitive impairment, aggression, and weight loss [1]. It is believed that the Htt genes, which are responsible for HD, have spread worldwide [2]. According to the global prevalence rate in 2017, 14 people per 100,000 are affected by HD each year, with a gender-related prevalence ratio of 13 women to 14 men. The prevalence rate was determined to be 66,787 after research was conducted in 2020 in eight different countries [3,4].

Huntington’s disease (HD) is characterised by an abnormally high amount of polyglutamine (poly Q) in the huntingtin (Htt) protein due to a triple repeat of cytosine, adenine, and guanine (CAG). CAG is typically repeated 35 times; however, in HD patients, the frequency rises from 36 to 39 times. CAG repetition is caused by a huntingtin (mHtt) gene mutation [5]. HD is a multisystem disorder because it affects the striatum, basal ganglia, cerebral cortex, and hippocampus [6]. The striatum and the cortex both suffer from the degeneration of neurons, followed by HD [7]. The brain’s striatum loses GABAergic medium spiny neurons (MSN), and the cortex experiences neurodegeneration, which alters mitochondrial function, inflammatory markers, oxidative stress, and neurotransmitters [8]. The specific mechanism by which neurons die in HD is unknown. HD causes damage to the brain’s thalamus, hypothalamus, subthalamic nucleus, substantia nigra (SN), and cerebellar nuclei [9,10]. Patients with HD may encounter cognitive problems; behavioural changes are frequently the most unpleasant feature of the disorder for patients and relatives [11]. Motor symptoms like “restlessness” and chorea, an irregular involuntary movement originating from the Greek word “dance,” are the most obvious HD symptoms [12]. HD is distinguished by quick, abrupt, irregular, unpredictable, and non-stereotypical movements [13]. The mitochondrial neurotoxin 3-NP inhibits succinate dehydrogenase, a TCA cycle enzyme, and the electron transport chain enzyme irreversibly [14,15]. Endogenously produced superoxide and hydroxyl free radicals can damage lipids, proteins, and DNA, altering brain structure, function, neurotransmitter, and inflammatory levels [16]. Reactive oxygen species (ROS) have been identified as potential mediators of neuronal cell death in HD [17]. Systemic administration of 3-NP results in CNS lesions that selectively target MSN in the striatum, reflecting HD’s spatial and neuronal selectivity [18]. 

The neurotoxin 3-NP impairs cognition and induces motor abnormalities such as dystonia, involuntary jerky movements, torsion spasms, and facial grimaces [19]; it is useful for determining the neuronal susceptibility and motor impairments associated with HD [20]. Striatum and brain lesions induced by 3-NP, together with increasing lactate levels, increased NMDA-receptor activation in rats, and this effect can be mitigated by employing NMDA-receptor antagonists [21]. Several investigations have linked 3-NP toxicity to increases in striatal hydroxyl (OH-) and superoxide (O_2_•-) free radical generation and oxidative damage indicators in the CNS [22]. Oxidative stress and NMDA enhance neuroinflammation and raise or activate proinflammatory markers (TNF-α and IL-1β) [23]. Increased DNA fragmentation and decreased apurinic or apyrimidinic endonuclease synthesis [24,25] indicate that older rats are more vulnerable to the oxidative stress generated by 3-NP. Following 3-NP injection, striatal glutathione (GSH), catalase (CAT), and superoxide dismutase (SOD) levels were reduced, indicating deficiencies in antioxidant mechanisms [26].

The bark of the *Boswelliaserrata* tree, usually found in the Burseraceae family, is used to make the gummy dry oleo-resin exudate. β-BA has been shown to decrease 5-lipoxygenase (5-LO), topoisomerases, angiogenesis, leukotrienes, and arachidonic acid, all known to inhibit proinflammatory and inflammatory cytokines [27]. β-BA potentially inhibits ERK and MAPK to protect the suprachiasmatic nucleus (SCN) following excessive glutamate release [28] or catalyse oxidation via the cytochrome p450 enzyme [27]. BAs and their derivatives have also exhibited anticarcinogenic, anti-hyperlipidemic, anti-atherosclerotic, anti-ulcer, wound-healing, analgesic, and antihyperglycemic activities [29,30]. According to the research, β-BA can modulate mitochondrial complex dysregulation by decreasing ROS production in stroke [31], Parkinson’s [32], and cancer [33], which is a major component in the formation of oxidative stress. In Alzheimer’s disease (AD), β-BA also participates in neurogenesis by balancing Ach and AchE [34]. Multiple sclerosis (MS) [35] and traumatic brain injury (TBI) [36] are two conditions wherein BA lowers glutamate levels, which results in excitotoxicity and oxidative damage to neuronal cells. Memory is also enhanced by β-BA because it increases the number of pyramidal cells in the striatum and the cortex [37].

Beta-tocopherol, also known as vitamin E, is an antioxidant that protects against damage to lipids. Vitamin E has been shown to reduce reactive oxygen species (ROS), inflammation, and oxidative damage, all of which may contribute to neuronal and neurochemical function recovery [38]. Vitamin E is used as a supplement, therapy, and standard medicine in various interventions due to its neuroprotective effect on CNS illnesses such as Alzheimer’s, Parkinson’s, ALS, and stroke [39]. A limited range of medications is available for managing HD symptoms. All of these findings, together with the lack of pharmacological intervention, urge the investigator to conduct an experiment to evaluate the efficacy of β-BA against 3-NP-induced HD in rats.

## 2. Material and Methods

### 2.1. Experimental Animals

The study used male Wistar rats that were 8–9 months old and weighed 220–250 g. The animals utilised in the experiment were obtained from the Institute’s Central Animal House. Polycyclic cages with wire mesh tops and soft bedding were used to house the animals. The animals were kept under controlled conditions with a 12 h light–dark cycle, controlled temperature (22 ± 2 °C), controlled humidity (65–70%), and free access to food and water. The experimental protocol was approved by Institutional Animal Ethics Committee (IAEC) as per the guidelines of the committee for the purpose of control and supervision of experiments on animals (CPCSEA), Government of India (573/PO/Re/S/02/CPCSEA). Animals were acclimatized to laboratory conditions prior to research.

### 2.2. Drugs and Chemicals

The 3-nitropropionic acid (3-NP) was purchased from Sigma-Aldrich (St. Louis, MO, USA). β-BA was provided as an ex gratia sample from BAPEX, Rajasthan. Analytical-grade versions of all other compounds were also employed in the investigation. Drug and chemical solutions were freshly prepared before administration to the rats. The recommended dosage of 3-NP is 10 mg/kg intraperitoneally (i.p.), given for 15 days. The saline solution contained 5% DMSO and had a pH of 7.4. For oral use, β-BA was dissolved in water (with 2% ethanol) (p.o.). As a standard drug, vitamin E (Evion^®^ 200 marketed formulation, manufactured by Merck Limited, New York City, NY, USA, manufacturing date: May 2018 and expiry date: July 2020) was dissolved in water (with ethanol) and administered by the oral route (p.o.).

### 2.3. Experimental Grouping of Animals

**Figure 1:**
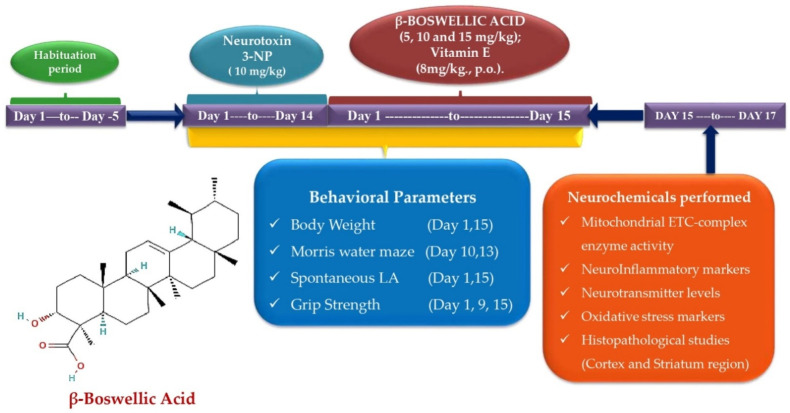
Behavioural and neurochemical data estimation protocol schedule.

The total number of days of the experiment was 15. A total of 42 Wistar male rats aged 8–9 months with a body weight of 220–250 g were required for conducting the protocol schedule. Rats were placed in polycyclic cages with wire mesh tops along with the soft, sterilized and residue-free wood saving with free food and water access. The animals were kept under controlled conditions with a 12 h light–dark cycle, controlled temperature (22.2 °C), controlled humidity (65–70%), and free access to food and water. The effect of the circadian rhythm was minimized by performing experiments from 9:00 a.m. to 1:00 p.m. The researcher performed an unblinded preclinical study on rats. Animals were randomly divided into seven groups as follows: Group 1: normal control; Group 2: Βeta-BOSWELLIC ACID (15 mg/kg/day, p.o.) perse; Group 3: 3-NP control (10 mg/kg/day, i.p.); Group 4: Βeta-Boswellic ACID (5 mg/kg/day, p.o.) + 3-NP (10 mg/kg/day, i.p.); Group 5: Βeta-Boswellic ACID (10 mg/kg/day, p.o.) + 3-NP (10 mg/kg/day, i.p.); Group 6: Βeta-Boswellic ACID (15 mg/kg/day, p.o.) + 3-NP (10 mg/kg/day, i.p.); Group 7: Vitamin E (8 mg/kg, p.o.) + 3-NP (10 mg/kg/day, i.p.) (Figure 1).

### 2.4. Measurement of Body Weight

Each rat’s body weight was measured on the first and last days of the protocol schedule. Body weight was calculated to evaluate changes in food and water intake in rats following the administration of 3-NP, along with various dosages of β-BA and conventional medication (vitamin E). Body weight was calculated as the percentage of change in the body weight of rats from the 1st day of the protocol to the last day, i.e., the 15th day of the treatment [40].

### 2.5. Parameters of Behavioral Assessment

#### 2.5.1. Morris Water Maze Test (MWM)

The Morris water maze apparatus was used to assess spatial learning and memory with cognition by observing rats’ performance efficiency. ELT and TSTQ tasks were conducted on the 10th, 13th, and last days of the protocol schedule. ELT refers to finding the platform in an MWM within a certain amount of time (in seconds) by rats. On the last day of the protocol, a hidden platform was put in a certain quadrant, and the rats were free to swim in the MWM for 120 s so that the TSTQ task could test how well their memories had been consolidated [41].

#### 2.5.2. Spontaneous Locomotor Activity (LA)

The actophotometer was used to assess spontaneous locomotor movements and motor coordination. The LA of each rat was analysed on the 1st and 15th days of the protocol schedule. The LA was measured for 5 min after the animal was placed in the actophotometer, and the value was stated as the number of beams crossed by the animal in 5 min [42].

#### 2.5.3. String Test for Grip Strength

A grip strength test was used to assess the ability of the forepaws to grasp and hold a wire (Hague Ave., Columbus, OH, USA). The grip strength test was performed on the 1st and 15th days of the procedure schedule. The latency of the rats’ paws to hold the wire against the researcher’s force was measured in terms of fall time, and the retaining latency on the wire was used to measure the rats’ gripping strength. Kilogram-force (KgF) is the force measurement unit used to quantify grip strength [43].

### 2.6. Biochemical Assessment of Parameters

#### 2.6.1. Biological Samples Preparation

The animal’s brain was removed with the decapitation method on the sixteenth day of the protocol schedule. The rat’s brain was cleaned with an ice-cold isotonic saline solution. Brain slices were homogenized and then put into a cold phosphate buffer solution of 0.1 M (7.4 pH). The homogeneous mixture was centrifuged at 10,500× *g* for 15 min to separate the supernatant. The biochemical changes (oxidative stress, neurotransmitter, neurochemical, cellular, and molecular) that happened in the brain after treatment with 3-NP and different doses of β-BA, in addition to the standard drug, were studied using a brain sample (supernatant) [44]. 

#### 2.6.2. Analyse the Enzyme Activity of Mitochondrial ETC Complexes in Rat Brain Homogenate

##### Post-Mitochondrial Supernatant (PMS) Preparation

The activity and concentration of the mitochondrial ETC-complex enzymes in rat brain homogenate were assessed by PMS. To prepare PMS, homogenate the entire brain and centrifuge at 5000 rpm for 20 min at 4 degrees Celsius to extract the supernatant. Pellets produced during PMS synthesis were combined in a 1:10 ratio with 0.1 M sodium phosphate buffer (pH 7.4) and gently stirred for 60 min at 4 °C. After centrifuging the mixture at 16,000× *g* for 30 min at 0 °C, it was resuspended in the same buffer. Add 250 mmol/L of additional sucrose to the resuspended pellet. After three rounds of centrifugation, enough buffer sucrose solution was produced to extract the mitochondrial fraction for further analysis [45].

##### Analyse the Enzyme Activity of Mitochondrial Complex-I (NADPH Dehydrogenase) Protein in Rat Brain Homogenate

The activity of the complex I enzyme was determined by oxidising NADH in an assay medium at 340 nm for 3 min at 37 °C. Rotenone exhibited sensitivity due to the complex I enzyme when 2 mM rotenone molecule reacted with complex I, and its activity was expressed in nM/mg protein [46].

##### Analyse the Enzyme Activity of Mitochondrial Complex-II (Succinate Dehydrogenase/SDH) Protein in Rat Brain Homogenate

The activity of the complex II enzyme was determined by combining homogenate gradient fraction (50 L) with sodium succinate solution (0.3 mL) and spectrophotometrically quantifying the absorbance at 490 nm (Shimadzu, UV-1700, Kyoto, Japan). The results were calculated using the chromophore molar extinction coefficient (1.36104 M^−1^ cm^−1^) and given as reduced INT. The values were given in nM/mg protein [47].

##### Analyse the Enzyme Activity of Mitochondrial ETC Complex-V (ATP Synthase) Protein in Rat Brain Homogenate

Sonication processes were conducted with the help of ice-cold perchloric acid (0.1 N) utilised on the aliquots of the homogenate aliquots to quickly inactivate the ATPase. Sonicate the homogenate, and then centrifuge it at 14,000× *g* for 5 min at 4 °C. The supernatant was collected, then neutralised with 1 N NaOH and preserved at −80 °C for further analysis. The amount of ATP in the supernatant was measured by reverse-phase HPLC (Perkin Elmer). The standard was dissolved, and the absorbance at 254 nm was used to make a standard ATP reference solution [48].

#### 2.6.3. Analysis of Neuroinflammatory Biomarkers

##### TNF-α and IL-1β Protein Concentration in the Brain

Inflammatory markers such as TNF-α and IL-1β levels in rat brain homogenate were determined using the ELISA kit from E-EL-R0019/TNF-α and E-EL-R0012/IL-1β Elabscienes (Wuhan, China). The activity of inflammatory indicators was represented as pg/mg protein [49,50].

#### 2.6.4. Analysis of Neurotransmitters

##### Dopamine Concentration in Rat’s Brain Homogenate

Striatal tissue samples were used to determine the dopamine levels in brain homogenates. Assess the DA level by using an electrochemical detector (ECD) and high-performance liquid chromatography (HPLC) (ECD Waters, Milford, MA, USA) in rat brain homogenates. The mobile phase was buffered with the help of sodium citrate (pH 4.5) and acetonitrile (87:13, *v*/*v*). The preparation of sodium citrate buffer was performed by the addition of citric acid (10 mM), NaH_2_PO_4_ (25 mM), EDTA (25 mM), and 1-heptane sulfonic acid (2 mM). A voltage of 0.75 V was used to conduct the electrochemical condition of the experiment and maintain its range from 5 to 50 nA. The separation was performed by using a 0.89 mL/min flow rate. A sample (20 mL) was manually injected into the injector. Homogenize the rat brain sample with centrifugation at 12,000× *g* for 5 min after adding 0.2 M perchloric acid to the homogenising solution. Dopamine levels were expressed in ng/mg [51].

##### GABA and Glutamate Concentration in Rat’s Brain Homogenate

The GABA and glutamate concentrations were determined using HPLC connected to an ECD. A Waters standard system, which includes a high-pressure isocratic pump, a 20 L manual sample injector valve, a C18 re-versed-phase column, and a UV detector, was used in conjunction with an ECD. The mobile phase was composed of 22% methanol, 25 mM EDTA, and 100 mM anhydrous disodium hydrogen phosphate (pH: 6.5). Experiments were performed with an electrochemical potential of +0.65 V and a range of sensitivity from 5 to 50 nA. The separation was conducted at a 1.2 mL/min flow rate with a 40 °C column temperature. Manual sample injections (20 L) were performed using a rheodyne valve injector. The frozen brain samples were thawed and mixed together with 0.2 M perchloric acid on the day of the experiment. Afterwards, the samples were subjected to 12,000 g of anteroposterior force for 15 min. When the supernatant was filtered through 0.22 m nylon filters and derivatized with OPA/-ME (o-pthalaldehyde/-mercaptoethanol), it was ready to be injected into the HPLC sample injector. Waters HPLC provided Breeze, version 3.2, which was used to record and analyse the data. We were able to calculate the concentrations of the amino acids of interest by extrapolating a standard curve with a concentration standard of between 10 and 100 ng/mL. Values were given in terms of ng/mg of protein, and a normal control group was used for comparison [52]. 

#### 2.6.5. Analysis of Oxidative Stress Parameters

##### Acetylcholinesterase (AChE) Concentration in Rat’s Brain Homogenate

A test was performed to figure out how much acetylcholine esterase was in the brain homogenate. A total of 3 mL of phosphate buffer (pH-8) and 0.05 mL of supernatant were mixed with 0.10 mL of DTNB and 0.10 mL of acetylthiocholine iodide. Spectrophotometry was used to measure the instantaneous changes at 420 nm, and the amount of AChE in the supernatant was calculated in terms of M/mg protein [53].

##### Malondialdehyde (MDA) Concentration in Rat’s Brain Homogenate

The Wills method was used to assess the end product of lipid peroxidation, MDA. Thiobarbituric acid was used to process the brain homogenate supernatant before 532 nm spectrophotometric measurement. MDA levels were expressed as nm/mg of protein [54].

##### Glutathione (GSH) Concentration in Rat’s Brain Homogenate

This assay was used to quantify the reduced glutathione level in rats’ brains homogenate after I.P. administration of 3-NP. The cold digestion process was performed with 1 ml supernatant in 4% sulfosalicylic acid (1 mL) at 4 °C for one hour, and then the collected sample was centrifuged at 1200 rpm for 15 min. In 1 mL supernatant, add 0.2 mL of DTNB and 2.7 mL phosphate buffer of 0.1 M concentration at pH 8. After the process, spectrophotometric measurements generated a yellow colour at the immediate level of 412 nm. The level of reduced GSH was expressed as M/mg protein [55].

##### Superoxide Dismutase (SOD) Concentration in Rat’s Brain Homogenate

The same method was used for epinephrine auto-oxidation at pH 10.4, spectrophotometrically assessing the level of SOD. The procedure began with the addition of 0.8 mL of pH 10.4 50 mM glycine buffer and 0.02 mL of epinephrine to the 0.2 mL of supernatant. After 5 min of incubation, the absorbance was taken at 480 nm. The SOD activity was reported at nM/mg of protein [56].

##### Catalase (CAT) Concentration in Rat’s Brain Homogenate

The concentration of CAT in rat brain homogenate can be measured by mixing 0.1 mL of supernatant with 1.9 mL of 50 mM phosphate buffer in a cuvette while keeping the pH at 7. After adding 1.0 mL of a freshly made 30 mM H_2_O_2_ solution, the reactions were started. Spectrophotometric analysis at 240 nm was used to determine the rate of H_2_O_2_ decomposition. The data were presented as micromoles of µM/ H_2_O_2_ decomposition per minute [57].

### 2.7. Histopathology

Following the end of the experimental procedure, the rats were anaesthetized with a 270 mg/kg i.p. dosage of sodium barbiturates. Decapitate the rats, then carefully separate their whole brains and wash them in PBS. Whole brains were preserved by being immersed in PBS containing 4% paraformaldehyde at a pH of 7.4. Afterwards, a microtome was used to cut sections of brain wax block that were between 4 and 5 mm in thickness. Hematoxylin and eosin staining and fluorescence microscopy (MOTICAM-Ba310 image plus 2.0, Schertz, TA, USA) at 100× magnification were used for a morphological study of brain sections (Cortex and striatum). At 100× magnification, different cell characteristics were seen in a Moticam, such as the size of the cell, apoptotic cells, inflammatory cells, and vacuolization in the cortex and striatum [58].

### 2.8. Statistical Analysis

Using one- and two-way ANOVA with post-hoc tests, the data was analysed and displayed through the analysed intervention. The data from the several experimental groups were separated and compared using Tukey’s multi-comparison test and Bonferroni’s test, respectively. Two-way ANOVA with Bonferroni’s posthoc test was performed to examine the behavioural changes (body weight, ELT, spontaneous LA, and grip strength) that occurred in rats after treatment. While neurochemical (estimation of mitochondrial complex activity, inflammatory biomarkers, neurotransmitter levels, and oxidative stress indicators) and behavioural alterations were evaluated using one-way analysis of variance (ANOVA) followed by Tukey’s post hoc test for multiple comparisons (TSTQ time). At a *p*-value of 0.01, the findings are considered statistically significant. The Kolmogorov–Smirnov test was utilised to ascertain the normal distribution of the sample size. The statistical analysis and visualization of the data were performed in the form of mean and standard deviation (SD) using GraphPad Prism version 5.03 for Windows (GraphPad Software, San Diego, CA, USA). 

## 3. Results

The study aimed to investigate the possible protective effect of β-B Ain HD. The therapeutic effects were evaluated using initial and final weight differences between animals in the control, inducing agent, and test control groups. Behavioural parameters like memory and learning were tested by a Morris water maze (MWM); for motor coordination grip strength (Rota-rod) model was used; for locomotor activity, an actophotometer was used; by histopathological examination of the brain tissue, tissue necrosis in the striatum and cortex was compared.

### 3.1. β-Boswellic Acid Ameliorates the Decreased Body Weight in 3-NP-Treated Rats

**Figure 2:**
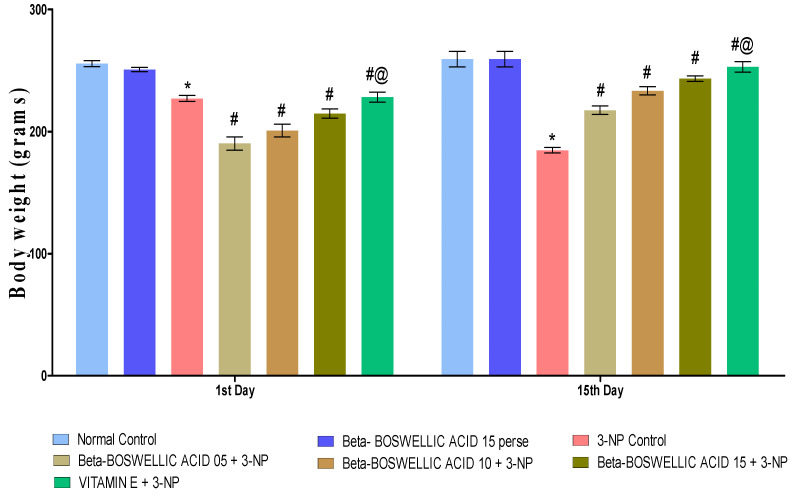
β-Boswellic acid ameliorates the decreased body weight in 3-NP-induced HD in rats. The Bonferroni two-way ANOVA post-hoc test was used to express significant statistical data as mean ± SD (*n* = 6) and * 3-NP (*p* < 0.01) versus normal and β-BA perse; # β-BA5 + 3NP, β-BA10 + 3NP, and β-BA15 + 3NP (*p* < 0.01) versus 3-NP; #@ Vitamin E + 3-NP (*p* < 0.01) versus β-BA5 + 3NP, β-BA10 + 3NP, and β-BA15 + 3NP.

Body weight was measured on the 1st and 15th days of the procedure schedule. Rats had significantly decreased body weight following chronic 3-NP treatment compared with the normal and perse groups. Normal and β-BA15 perse groups did not show any significant changes. Different doses of β-BA (5, 10, and 15 mg/kg) were shown to be more effective in treating rats than the 3-NP-induced HD group [two-way ANOVA:F (6, 70) = 123.13, *p* < 0.01] (Figure 2). In conventional medicine, vitamin E was shown to significantly improve body weight regulation compared to different doses of β-BA.

### 3.2. Behavioural Parameters

These behavioural (MWM, grip strength, locomotor activity) parameters were used to explore the neuroprotective effects of β-BA at the doses of 5, 10 & 15 mg/kg, p.o. compared with vitamin E. 

#### 3.2.1. β-Boswellic Acid Ameliorates Spatial Navigation Task in 3-NP Treated Rats

**Figure 3:**
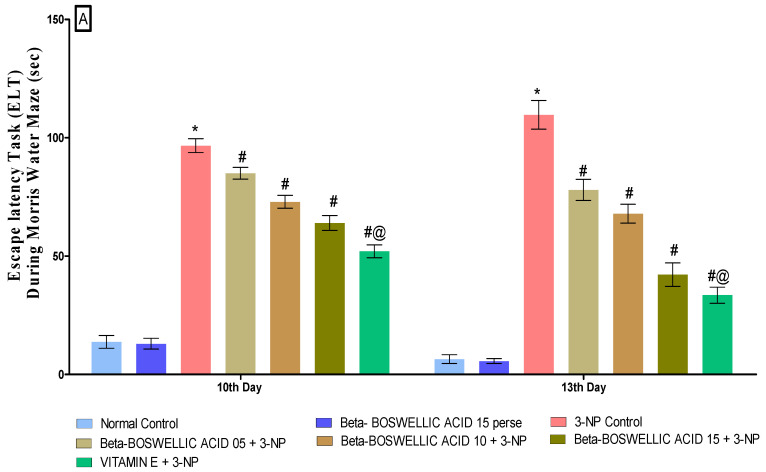

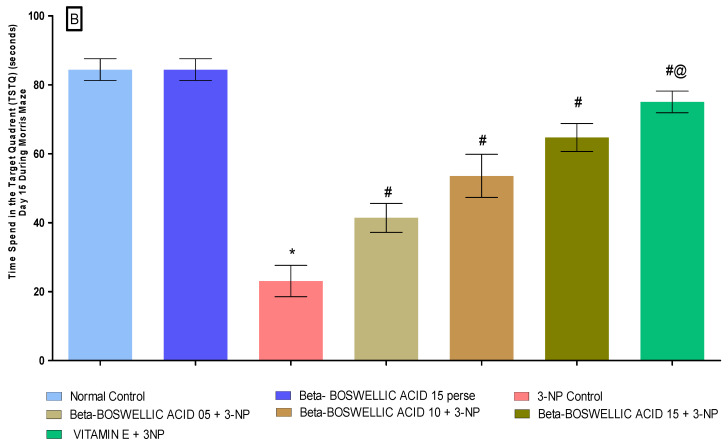
(**A**): β-Boswellic acid ameliorates ELT on Morris water maze in 3-NP-induced HD in rats. (**B**): β-Boswellic acid ameliorates TSTQ on Morris water maze in 3-NP-induced HD in rats. The Bonferroni Two-way ANOVA and Tukey’s one-way ANOVA post-hoc test was used to express significant statistical data as mean ± SD (*n* = 6); * 3-NP (*p* < 0.01) versus normal and β-BA perse; # β-BA5 + 3NP, β-BA10 + 3NP, and β-BA15 + 3NP (*p* < 0.01) versus 3-NP; #@ vitamin E + 3NP (*p* < 0.01) versus β-BA5 + 3NP, β-BA10 + 3NP, and β-BA15 + 3NP.

Spatial navigation tasks were conducted on the 10th and 13th days of the trial, according to the protocol. These two tasks, the escape latency task and time spent in the targeted quadrant (TSTQ), were performed by rats for the spatial navigation task. TSTQ was performed on the 13th day of the protocol schedule, while ELT was performed on the 10th and 13th days. There were no significant differences observed between the normal and perse groups. Chronic administration of 3-NP significantly increased ELT time and decreased TSTQ compared with the normal and β-BA15 perse group. Treatment with different doses of β-BA (5, 10, and 15 mg/kg) resulted in a substantial improvement in ELT [two-way ANOVA:F (6,70) = 31.88, *p* < 0.01] (Figure 3A) and TSTQ [one-way ANOVA:F (6, 30) = 1.548, *p* < 0.01] (Figure 3B) in rats compared with the 3-NP-induced HD. Furthermore, compared with the varying doses of β-BA and the standard medicine, vitamin E showed a considerable role in reducing ELT duration and elevating TSTQ.

#### 3.2.2. β-Boswellic Acid Ameliorates Locomotor Activity in 3-NP-Treated Rats 

**Figure 4:**
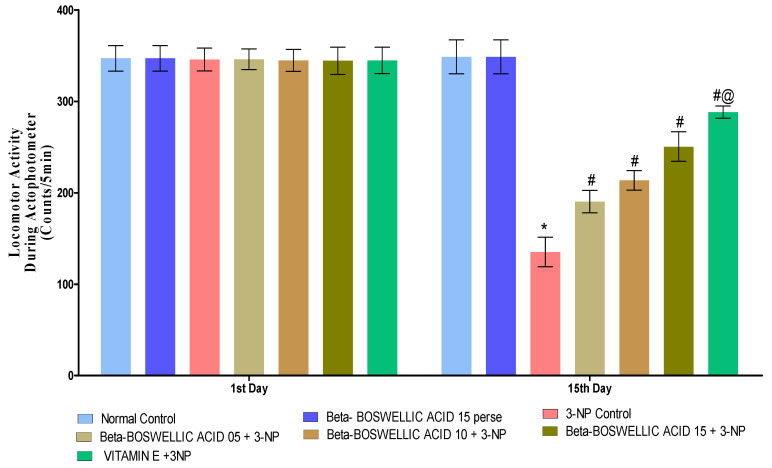
β-Boswellic acid ameliorates locomotion activity on actophotometer in3-NP-induced HD in rats. The Bonferroni two-way ANOVA and Tukey’s one-way ANOVA post-hoc test was used to express significant statistical data as mean ± SD (*n* = 6); * 3-NP (*p* < 0.01) versus normal and β-BA perse; # β-BA5 + 3NP, β-BA10 + 3NP, and β-BA15 + 3NP (*p* < 0.01) versus 3-NP; #@ vitamin E + 3NP (*p* < 0.01) versus β-BA5 + 3NP, β-BA10 + 3NP, and β-BA15 + 3NP.

On the 1st and 15th days of the experimentation schedule, locomotor activity (LA) was carried out to quantify the effect on the movement of β-BA alone and in combination with the standard drug, i.e., vitamin E. Neither the first nor fifteenth day showed any significant difference between the normal and β-BA15 perse groups. When 3-NP was administered to rats for the first time on day 1, no major changes were noticed when compared with the normal and perse groups. However, substantial differences were observed when 3-NP was chronically given over the course of 15 days. A series of β-BA (5, 10, and 15 mg/kg doses) showed a significant improvement in the locomotor activity against 3-NP-induced HD in rats [Two-way ANOVA:F (6, 70) = 97.34, *p* < 0.01] (Figure 4). On the 15th day of the trial, conventional medication, i.e., vitamin E, showed a considerable restoration in locomotion compared to different dosages of β-BA on the 15th day of the trial.

#### 3.2.3. β-Boswellic Acid Ameliorates Grip Strength in 3-NP-Treated Rats

**Figure 5:**
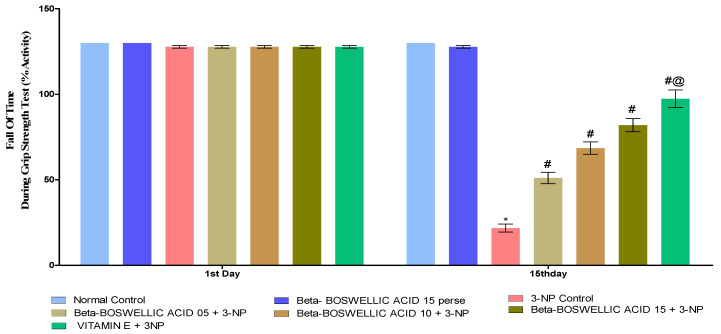
β-Boswellic acid ameliorates grip strength during grip strength task 3-NP-induced HD in rats. The Bonferroni two-way ANOVA and Tukey’s one-way ANOVA post-hoc test was used to express significant statistical data as mean ± SD (*n* = 6); * 3-NP (*p* < 0.01) versus normal and β-BA perse; # β-BA5 + 3NP, β-BA10 + 3NP, and β-BA15 + 3NP (*p* < 0.01) versus 3-NP; #@ vitamin E+ 3NP (*p* < 0.01) versus β-BA5 + 3NP, β-BA10 + 3NP, and β-BA15 + 3NP.

The grip strength task was completed three days after the protocol schedule (1st and 15th). We found no statistically significant differences between the control and β-BA15 perse groups. On the first day following the administration of 3-NP, no significant differences were seen when compared with normal and perse. However, during the chronic treatment of 3-NP, the HD rat’s grip strength significantly decreased on day 15. Grip strength was significantly enhanced in HD rats after they were treated with a series of doses of β-BA [two-way ANOVA:F (12, 105) = 686.96, *p* < 0.01] (Figure 5). When we compared different doses of β-BA and conventional vitamin E, we found that they improved grip strength. 

### 3.3. Biochemical Parameters

#### 3.3.1. β Boswellic Acid Ameliorates Mitochondrial ETC Complexes Activity in 3-NP Treated Rats

**Figure 6:**
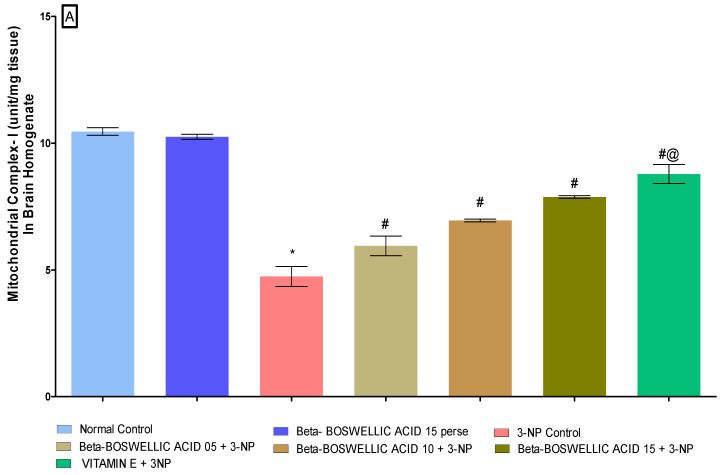

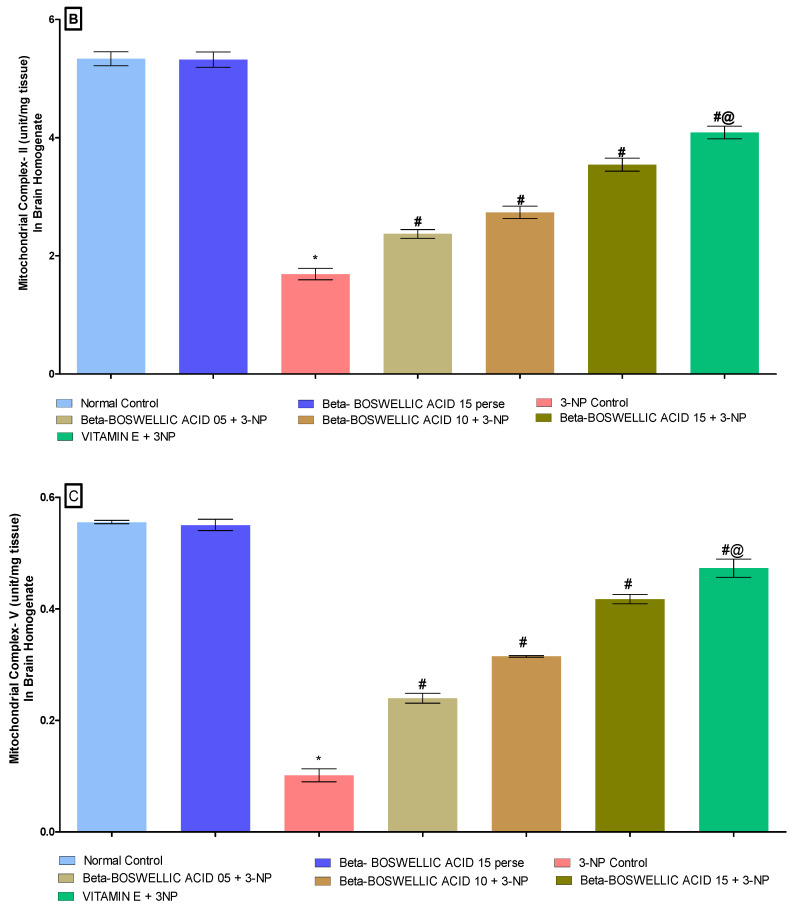
(**A**–**C**): β-Boswellic acid ameliorates mitochondrial ETC complexes activity in brain homogenates of 3-NP-induced HD in rats. The Bonferroni two-way ANOVA and Tukey’s one-way ANOVA post-hoc test was used to express significant statistical data as mean ± SD (n = 6); * 3-NP (*p* < 0.01) versus normal and β-BA perse; # β-BA5 + 3NP, β-BA10 + 3NP, and β-BA15 + 3NP (*p* < 0.01) versus 3-NP; #@ vitamin E + 3NP (*p* < 0.01) versus β-BA5 + 3NP, β-BA10 + 3NP, and β-BA15 + 3NP.

The activity of mitochondrial enzyme complexes (Complex I, II, and V) in rat brain homogenates was quantified at the end of the protocol schedule. No discernible differences were seen between the normal and β-BA15 perse groups. Mitochondrial enzyme activity was shown to significantly decrease following repeated 3-NP treatment. A substantial increase in mitochondrial enzyme complex I [One-way ANOVA:F (6, 30) = 0.5016, *p* < 0.01] (Figure 6A), complex II [One-way ANOVA:F (6, 30) = 0.7639, *p* < 0.01] (Figure 6B) and complex V [One-way ANOVA:F (6, 30) = 0.4812, *p* < 0.01] (Figure 6C) activity was seen in HD rats after treatment with varying dosages (5, 10, and 15 mg/kg) of β-BA. Treatment with a standard drug, such as vitamin E, has been shown to boost mitochondrial enzyme activity and stop mitochondria from becoming dysfunctional.

#### 3.3.2. β-Boswellic Acid Ameliorates Inflammatory Markers (TNF-α,& IL-1β) Level in 3-NP Treated Rats

**Figure 7:**
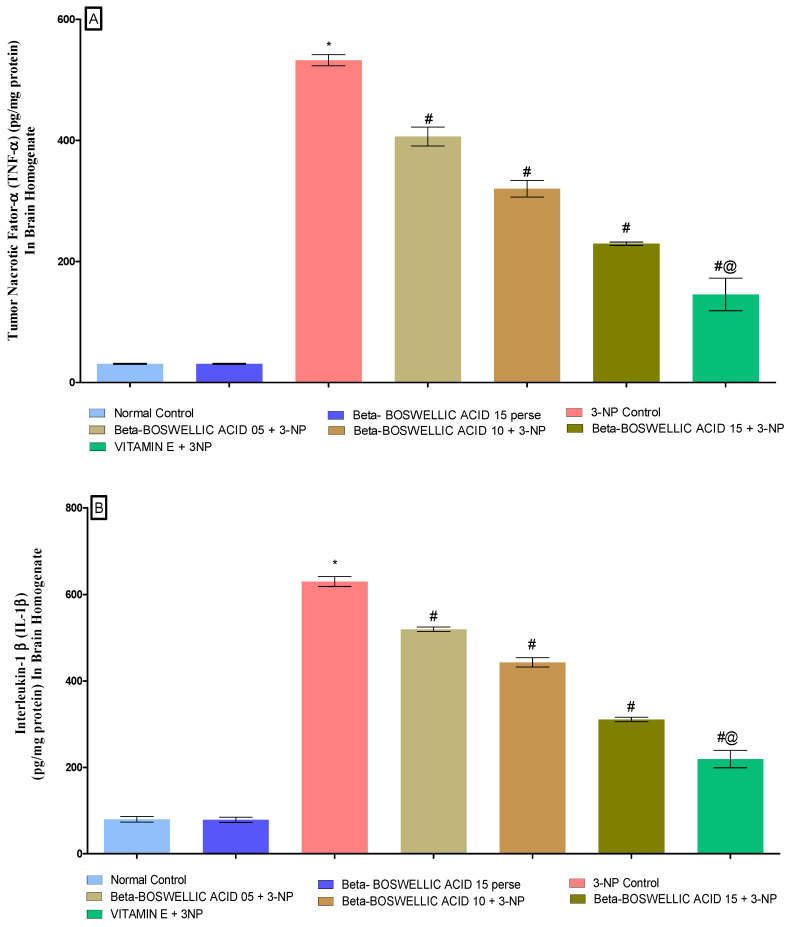
(**A**,**B**): β-Boswellic acid ameliorates inflammatory markers level in brain homogenates of 3-NP-induced HD in rats. The Tukey’s one-way ANOVA post-hoc test was used to express significant statistical data as mean ± SD (*n* = 6); * 3-NP (*p* < 0.01) versus normal and β-BA perse; # β-BA5 + 3NP, β-BA10 + 3NP, and β-BA15 + 3NP (*p* < 0.01) versus 3-NP; #@ vitamin E + 3NP (*p* < 0.01) versus β-BA5 + 3NP, β-BA10 + 3NP, and β-BA15 + 3NP.

We estimated the level of inflammatory markers (TNF-α and IL-1β) in the rat brain homogenates at the end of the treatment schedule. No statistically significant differences were found in either the normal or the perse group. When rats were given 3-NP for an extended period of time, inflammatory markers increased in comparison to the control and perse groups; however, when the animals were given β-BA, TNF-α [one-way ANOVA:F (6, 30) = 0.6244, *p* < 0.01] (Figure 7A) and IL-1β [one-way ANOVA:F (6, 30) = 0.1916, *p* < 0.01] (Figure 7B) levels were significantly reduced and improved in a dose-dependent manner. In rats with 3-NP-induced HD, standard drugs (like vitamin E) helped restore signs of inflammation. 

#### 3.3.3. β-Boswellic Acid Ameliorates Neurotransmitter Levels (Dopamine, Glutamate, and GABA) in 3-NP Treated Rats

**Figure 8:**
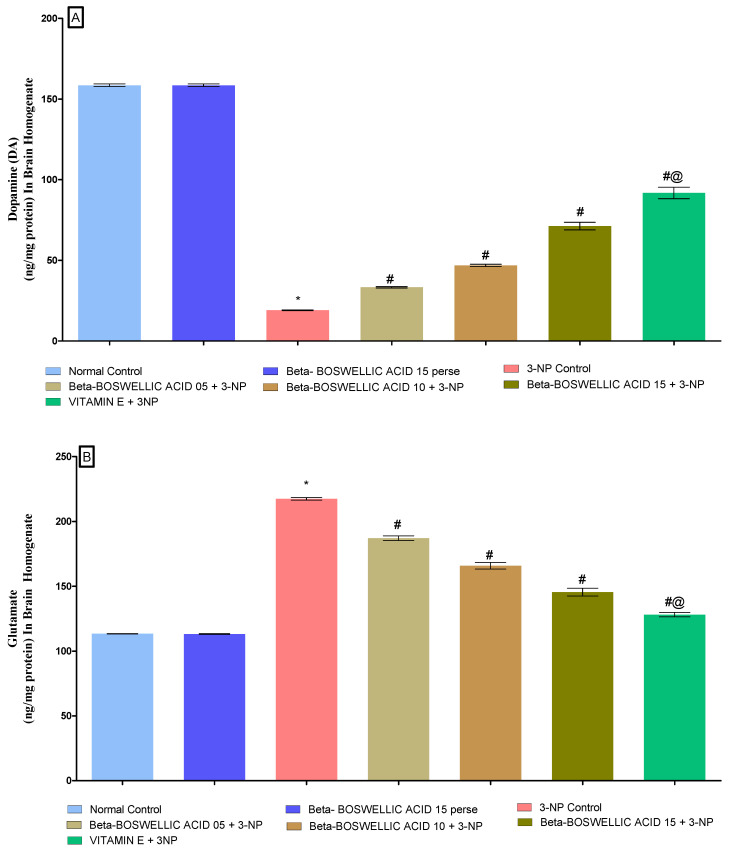

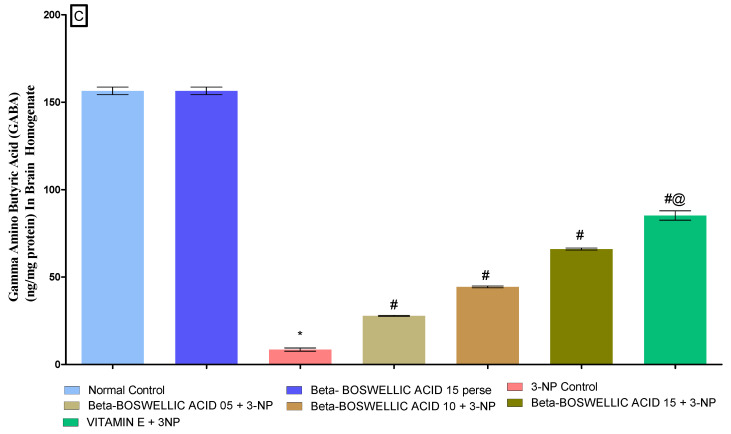
(**A**–**C**): β-Boswellic acid ameliorated neurotransmitter levels in brain homogenates of 3-NP-induced HD in rats. The Tukey’s one-way ANOVA post-hoc test was used to express significant statistical data as mean ± SD (*n* = 6); * 3-NP (*p* < 0.01) versus normal and β-BA perse; # β-BA5 + 3NP, β-BA10 + 3NP, and β-BA15 + 3NP (*p* < 0.01) versus 3-NP; #@ vitamin E + 3NP (*p* < 0.01) versus β-BA5 + 3NP, β-BA10 + 3NP, and β-BA15 + 3NP.

According to the protocol schedule, neurotransmitter levels were quantified in the brain homogenates of rats at the end of the protocol. There was no difference observed in the normal and β-BA15 perse groups. On the other hand, the chronic administration of 3-NP showed a significant decrease in dopamine (DA) and GABA levels while increasing glutamate levels in HD rats. However, a dose-dependent treatment of β-BA significantly alleviates DA [One-way ANOVA:F (6, 30) = 0.2963, *p* < 0.01] (Figure 8A) and GABA levels [One-way ANOVA:F (6, 30) = 1.016, *p* < 0.01] (Figure 8C), whereas it decreases glutamate levels [One-way ANOVA:F (6, 30) = 1.327, *p* < 0.01] (Figure 8B) of 3-NP treated rats. Standard treatment played a key role in restoring the level of neurotransmitters in rats that had been affected by 3-NP.

#### 3.3.4. β-Boswellic Acid Amelioratesoxidative Stress (AChE, MDA, Reduced GSH, SOD, and CAT) Parameters in 3-NP Treated Rats

**Figure 9:**
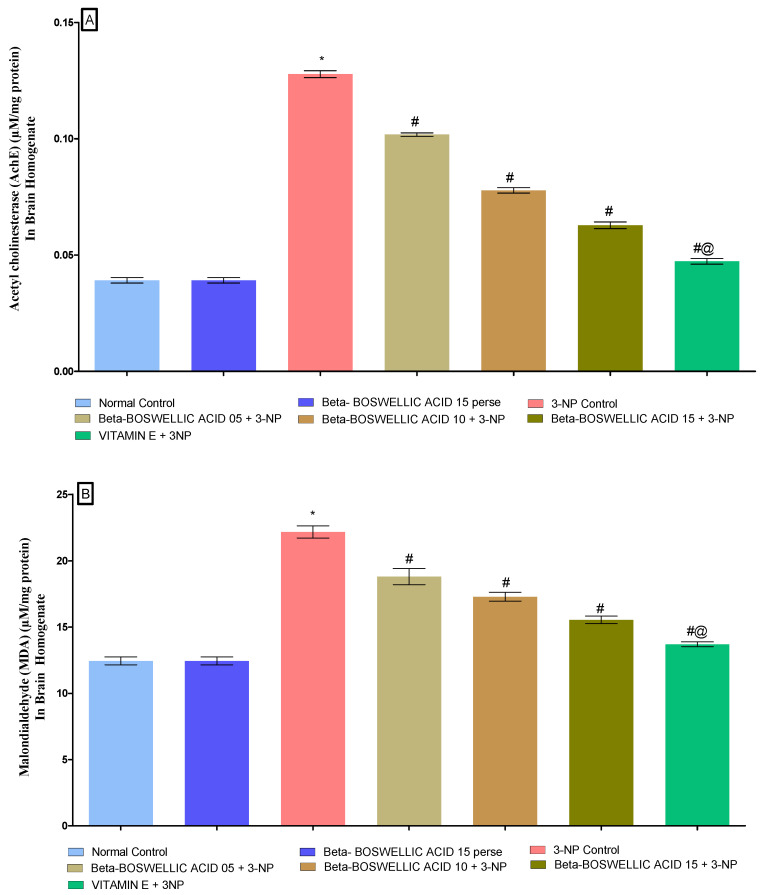

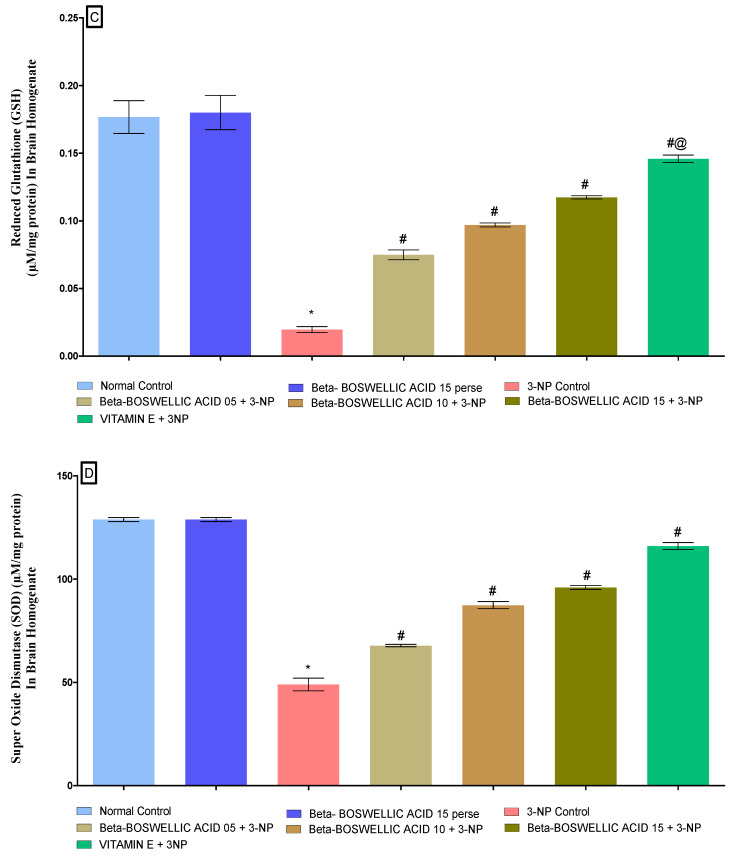

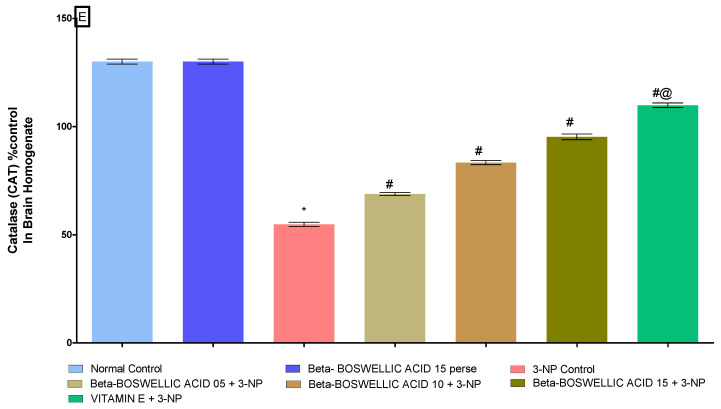
(**A**–**E**): β-Boswellic acid ameliorates the oxidative stress parameters in brain homogenates of 3-NP-induced HD in rats. The Tukey’s one-way ANOVA post-hoc test was used to express significant statistical data as mean ± SD (*n* = 6); * 3-NP (*p* < 0.01) versus normal and β-BA perse; # β-BA5 + 3NP, β-BA10 + 3NP, and β-BA15 + 3NP (*p* < 0.01) versus 3-NP; #@ vitamin E + 3NP (*p* < 0.01) versus β-BA5 + 3NP, β-BA10 + 3NP, and β-BA15 + 3NP.

At the end of the protocol, the levels of oxidative indicators in rat brain homogenates were measured; these included the enzyme acetylcholinesterase (AChE), as well as malondialdehyde (MDA), glutathione (GSH), superoxide dismutase (SOD), and catalase (CAT). No significant differences were found between the normal and β-BA 15 perse groups. However, significant differences were seen after the administration of 3-NP. β-BA also reduced AChE [one-way ANOVA:F (6, 30) = 1.175, *p* < 0.01] (Figure 9A), MDA [one-way ANOVA: F (6, 30) = 0.5999, *p* < 0.01] (Figure 9B), GSH [one-way ANOVA: F (6, 30) = 1.003, *p* < 0.01] (Figure 9C), SOD [One-way ANOVA:F (6, 30) = 1.195, *p* < 0.01] (Figure 9D), CAT [one-way ANOVA:F (6, 30) = 1.274, *p* < 0.01] (Figure 9E) level in a dose-dependent manner in 3-NP-induced HD in rats. Compared with the various doses of β-BA, the standard medicine vitamin E efficiently regulated the modulated level of oxidative markers. 

### 3.4. Histopathological Analysis

#### Effect of β-Boswellic Acid on 3-NP Induced Histopathological Changes in Rat Brain

Histological sections of the cortex and striatum from normal, β-BA- and vitamin E-treated rats revealed properly sized, intact pyramidal-shaped neuronal cells with a clearly visible cell nucleus without vacuolization and a continuous cell membrane without haemorrhage. After a prolonged i.p. injection of 3-NP to develop HD in rats, histopathological alterations were detected. The histological examination of a brain segment treated with 3-nitropropionic acid (cortex and striatum) revealed many neuronal spaces with fuzzy borders and haemorrhages in cerebral tissues. In contrast, the putamen and caudate revealed localized neurodegeneration and necrosis, cell feature loss, and pyramidal shape loss. 

**Figure 10:**
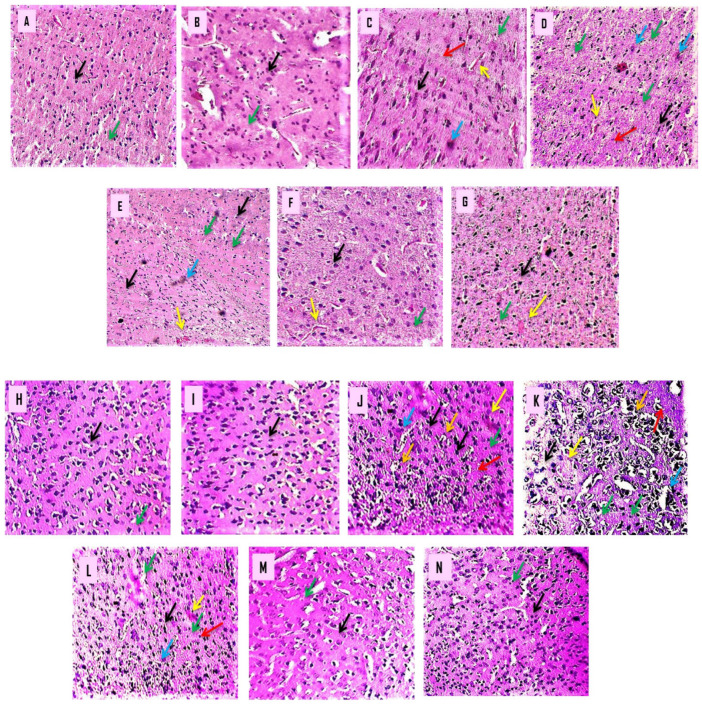
(**A**–**N**): β-Boswellic acid ameliorates the histopathological changes in the brain’s striatum and cortex region after administration of 3-NP in rats to induce HD. The goal of this experiment was to analyse histopathological information of the striatum and cortex sections by using H- and E-stain. After 3-NP delivery into a rat’s brain, striatal and cortical sections revealed the characteristic morphological alterations associated with HD: uneven, flattened cells; cells devoid of nucleus and membrane; bleeding; apoptosis; degeneration; gliosis; and vacuolization of cells. The red arrow represents neuronal cell degeneration, and apoptotic cells are shown with the blue arrow; cortical bleeding is shown with the yellow arrow; gliosis is shown with the green arrow; cell vacuolization is shown with the orange arrow, especially in the cortical section of the brain, and the black arrow displays the form of the cell. (**A**) This diagram depicts a typical, randomly assigned group of six rats characterized by a high neuronal cell density and a pyramidal shape. (**B**) It did not show a big difference between the β-BA perse group and the normal group in shape or cell density. (**C**) Schematic representation of the 3-NP-treated group, which had uneven and flattened cells, cells lacking a nucleus and membrane, haemorrhage, apoptosis, gliosis and vacuolization of the cell (cortical region). (**D**–**F**) Diagrams demonstrated that, when compared with the 3-NP-treated group, β-BA 5, β-BA 10, and β-BA 15 significantly reduced irregular and flattened cells, cells without nucleus and membrane, bleeding, apoptotic degeneration, gliosis and vacuolization of a cell (cortical region). (**G**) As shown in the diagram, the β-BA 5, β-BA 10, and β-BA 15 groups had a lower rate of restoring neurons than the vitamin E. (A magnification scale of 100× was used for morphological studies of brain sections such as the cortex and striatum).

β-BA15 mg/kg, p.o. supplementation significantly attenuated 3-NP-induced histological alteration as compared to β-BA5 mg/kg, p.o. and β-BA10 mg/kg, p.o. supplementation. When treated with 3-NP 10 mg/kg + β-BA5 mg/kg, p.o., caudate, and putamen showed focal neurodegeneration and necrosis with a loss of cell details, shape, and size. The caudate and putamen showed focal mild degenerative changes in treatment with 3-NP 10 mg/kg + β-BA10 mg/kg, p.o. The caudate and putamen showed mild focal gliosis in treatment with 3-NP 10 mg/kg + β-BA15 mg/kg, p.o. (Figure 10).

## 4. Discussion

Our hypothesis stated that -BA has a neuroprotective effect as a CAG triplet repeat inhibitor in 3-NP-induced HD rats by lowering mHtt gene expression and preventing neurodegeneration in medium spiny neurons (MSN). Neurochemical and behavioural alterations were seen after i.p. treatment of 3-NP to rats. We determined that this was due to the neurodegeneration of GABAergic medium spiny neurons (MSN) and CAG repetition in the striatum and cortex.

β-BA is a terpenoid composed of a pentacyclic chain of carbon chains [59]. -BA was synthesised using the herb Boswellia serrate [46]. β-BA has previously been found to be neuroprotective against streptozotocin-induced Alzheimer’s disease, to aid in mitochondrial malfunction recovery, and to regulate behavioural and neurochemical abnormalities [60]. In ALS rats, acetyl-11-keto-beta Boswellic acid improved body weight, memory and cognition, locomotion, and muscle incoordination [61,62]. Clinical data and research revealed that frequent weight loss occurs in the later stages of HD, resulting in chronic fatigue syndrome [63]. In juveniles and adults, 3-NP mimics and produces HD-like behaviour (weight loss, muscular wasting, and skeletal muscle atrophy) [64]. β -BA protects neurochemicals from damage by regulating inflammatory cytokines, neurotransmitters, and oxidative stress [65,66]. Moreover, in current investigations, vitamin E was used as a therapeutic intervention because of its antioxidative effects, and it also helps with neuroprotection [67]. According to our research findings, long-term treatment of 3-NP-induced HD resulted in decreased rat body weight. The return to a normal weight was assisted by both β-BA and vitamin E.

CAG repetition may cause muscle incoordination, muscle wasting, and skeletal muscle atrophy in HD patients [68]. Degeneration and lesion formation in rat cortical and striatal neurons delayed muscle movement and impaired coordination [69]. Chronic 3-NP injection promoted mitochondrial dysfunction, disrupting neurotransmitters that influence motor and muscular activity [21,70]. In our experiment, HD-induced rats treated with β-BA and 3-NP improved their locomotor activity and gripping muscle strength in a dose-dependent manner.

3-NP is a mitochondrial toxin [71,72] that inhibits succinate dehydrogenase (ETC complex II) function, leading to mitochondrial failure and an increase in the CGA repeating cycle, which results in the putamen and caudate neurodegeneration [73,74,75]. Neurodegeneration [76] and mitochondrial failure [77] affect morphology, behaviour, and neurochemistry.

The mitochondrial deficit may result in ROS, which increases oxidative stress [78,79] and lowers antioxidants, resulting in the progression of brain inflammation and proinflammatory cytokines, as well as the deterioration of HD [80]. The neurotoxin 3-NP changes rats’ neurochemistry, behaviour, and morphology after intraperitoneal administration. The neurotoxin 3-NP investigates HD using an animal model [81]. In this work, rats were fed 3-NP daily for 14 days to induce HD-like symptoms. Cognitive and motor impairment are clinical symptoms of Huntington’s disease [82]. Frontostriatal loop neurodegeneration increases glutamate levels, decreasing short-term working memory and causing abnormalities in learning behaviour [83]. Mitochondrial toxin 3-NP produces neurodegeneration in the frontostriatal loop, resulting in cognitive and memory deficits [84]. Our findings suggest that β-BA may benefit rats suffering from memory and cognitive impairments caused by long-term exposure to 3-NP.

Mitochondria are essential in the control of ROS generation and neuroprotective properties [85]. Mitochondrial malfunction increases ROS production and causes neuronal injury [86]. The neurotoxin 3-NP inhibits the activity of the ETC complexes I, II, and V [87,88]. The neurotoxin 3-NP may promote inefficient energy generation and accelerate disease progression. In the process of our investigation, we found that 3-NP caused HD to be produced in the brains of rats. Additionally, the mitochondrial ETC complexes I, II, and V enzyme levels were also determined in rat brain homogenates. Whereas β-BA and vitamin E significantly restored the ETC-complex enzyme level in brain homogenate samples. Proinflammatory cytokines (TNF-α, IL-1β) were activated in response to neurodegeneration and neurotoxicity, increasing the likelihood of disease [89]. Damage to GABAergic MSN caused by 3-NP (as reported in [19,90]) led to increased proinflammatory cytokines. We revealed that a high dose of β-BA significantly reduced the level of proinflammatory cytokines in 3-NP-exposed rats.

Patients with HD may experience changes in their levels of DA, glutamate, and GABA due to neurodegeneration caused by MSN [91]. The neurotoxin 3-NP changes neurotransmitter levels in rats and individuals with Parkinson’s disease [92]. We found that giving rats 3-NP generates neurotransmitter alterations comparable with those observed in HD patients, where a current investigation found that β-BA and vitamin E contributed to restoring the neurotransmitter levels.

Clinical findings show that HD patients have increased oxidative stress (AChEs and MDA) due to reduced antioxidant levels (GSH, SOD, and CAT) related to excessive ROS production [93]. Mitochondrial dysfunction is closely connected to aberrant ROS generation [94]. As previously stated, the mitochondrial toxin 3-NP modulates the amount of oxidative stress [95]. In our investigation, i.p. injections of 3-NP affected the levels of oxidative biomarkers in the striatum, and the cortex was restored by β-BA.

Degeneration of MSN neurons could be seen in the striatum and cortex of HD patients. In HD-induced rats’ brains, H- and E-staining revealed morphological abnormalities, apoptotic cells, cells losing nucleus and membrane, degenerative neurons, and gliosis [96]. The neurotoxin 3-NP caused neurodegeneration and was found in HD patients [97,98].

According to the findings of our research, administering high dosages of β-BA could reverse the histopathological change in 3-NP-toxicated HD rats. Based on the results of our research and the statistics, we believe that high-dose β-BA may be a more effective treatment for HD in further studies. However, the underlying technique must be confirmed using knock-in and knock-out procedures. Cellular and molecular marker studies such as Western blot and immunohistochemistry will almost definitely confirm these findings. According to our findings, conventional medications worked better when paired with beta Boswellic acid and could be used to treat Huntington’s disease. We also proposed an additional study to better understand the relationship between prescribed drugs and HD treatments.

## 5. Conclusions

In this investigation, we revealed that β-BA exhibited a dose-dependent neuroprotective effect compared with the conventional treatment of vitamin E after 3-NP i.p. injection-induced lesions and neurodegeneration in rats’ brains. Based on our histological findings, we concluded that a dose-dependent administration of β-BA acid could slow the progression of neurodegeneration as well as morphological change in the striatum and cortex. The neurotoxin 3-NP caused neurobehavioral dysfunctions, including motor and cognitive abnormalities, which could be reversed with β-BA therapy, providing additional protection against developing HD-like symptoms. Our findings strongly suggested that β-BA could reduce cytokine production while restoring mitochondrial ETC-complex enzymes, neurotransmitter imbalances, and antioxidant potential. In addition, β-BA has a role in restoring neurobehavioral, neurochemical, cellular, and disease-causing processes regulated by the striatum and the cortex region of the brain. All of these findings suggest that β-BA has clinical potential as a treatment for the pathogenesis of HD.

## Data Availability

All data generated or analysed during this study are included in this article. There are no separate or additional files.

## Abbreviations

ACh	Acetylcholine
AChE	Acetyl cholinesterase
AD	Alzheimer disease
ALS	Amyotrophic lateral sclerosis
ANOVA	Analysis of variance
ATP	Adenosine triphosphate
β-BA	Beta Boswellic acid
BG	Basal Ganglia
CAG	Cytosine, adenine, guanine
CAT	Catalase
CNS	Central nervous system
DA	Dopamine
DNA	Deoxyribonucleic acid
DTNB	5,5′-dithiobis-(2-nitrobenzoic acid)
ECD	Electron capture detector
EDTA	Ethylenediaminetetraacetic acid
ELISA	Enzyme-linked immunosorbent assay
ELT	Escape latency test
ETC	Electron transport chain
GABA	Gamma-amino butyric acid
GP	Globus pallidus
GSH	Glutathione
HD	Huntington diseases
H_2_O_2_	Hydrogen peroxide
HPLC	High-Performance Liquid Chromatography
Htt	Huntigtin gene
LA	Locomotor activity
5-LO	5-lipoxygenase
MDA	Malondialdehyde
MAPK	Mitogen-activated protein kinase
mHtt	Mutated huntigtin gene
MSN	Medium spiny neurons
MWM	Morris water maze
NMDA	N-methyl-D-aspartate
3-NP	3-Nitropropionic acid
PD	Parkinson’s disases
Poly Q	Polyglutamine
O_2_•	Superoxide
OH	Hydroxyl ion
ROS	Reactive oxygen species
SLA	Spontaneous locomotor activity
SN	Substantianigra
SOD	Superoxide dismutase
TCA	Trichloroacetic acid
TH	Tyrosine hydroxylase
TL	Transfer latency
TNF-α	Tumour necrosis factor-α
TSTQ	Time spent in the target quadrant zone
